# Systematic Analysis of Cellular Signaling Pathways and Therapeutic Targets for SLC45A3:ERG Fusion-Positive Prostate Cancer

**DOI:** 10.3390/jpm12111818

**Published:** 2022-11-02

**Authors:** Jongsu Kim, Kyung Won Hwang, Hye Jung Lee, Hong Sook Kim

**Affiliations:** Department of Biological Sciences, Sungkyunkwan University, Suwon 16419, Korea

**Keywords:** *SLC45A3*, *ERG*, prostate cancer, drug repurposing, gene fusion, TCGA

## Abstract

ETS-related gene (*ERG)* fusion affects prostate cancer depending on the degree of expression of *ERG*. Solute Carrier Family 45 Member 3 (*SLC45A3*) is the second-most common 5′ partner gene of *ERG* rearrangement. However, the molecular pathological features of *SLC45A3:ERG* (*S:E*) fusion and therapeutic methods have not been studied at all. *S:E* fusion-positive cancers (*n* = 10) were selected from the Tumor Fusion Gene Data Portal website. Fusion-negative cancers (*n* = 50) were selected by sorting *ERG* expression level in descending order and selecting the bottom to 50th sample. Totally, 1325 *ERG* correlated genes were identified by a Pearson correlation test using over 0.3 of absolute correlation coefficiency (|R| > 0.3). Pathway analysis was performed using over-representation analysis of correlated genes, and seven cancer-related pathways (focal adhesion kinase (FAK)/PI3K-Akt, JAK-STAT, Notch, receptor tyrosine kinase/PDGF, TGF-β, VEGFA, and Wnt signaling) were identified. In particular, focal adhesion kinase (FAK)/PI3K-Akt signaling and JAK-STAT signaling were significantly enriched in *S:E* fusion-positive prostate cancer. We further identified therapeutic targets and candidate drugs for *S:E* fusion-positive prostate cancer using gene–drug network analysis. Interestingly, *PDGFRA* and *PDGFRB* were the most frequently predicted therapeutic targets, and imatinib targeted both genes. In this study, we provide extensive information on cellular signaling pathways involved in *S:E* fusion-positive prostate cancer and also suggest therapeutic methods.

## 1. Introduction

Prostate cancer is a common diagnosis in males and is the second most common cancer causing mortality in males. More than 190,000 cases of prostate cancer were reported in the United States in 2020 [1]. ETS-related gene (*ERG*), a member of the E-26 transformation-specific (ETS) family, is an oncogene regulating cell proliferation, differentiation, and metastasis [2]. Previous studies found that ~55% of prostate cancer patients have *ERG* overexpression driven by fusion of the *ERG* gene with androgen response genes such as *TMPRSS2* [2,3]. *TMPRSS2:ERG* (*T:E*) fusion is most frequently found in prostate cancer, and its oncogenic role and regulating molecular mechanisms have been well studied [2,4,5]. Inhibitors or small molecules targeting the *T:E* fusion have been suggested but no drugs have been FDA approved to date [6]. In our previous study, the altered cellular signaling pathways in *T:E* fusion-positive prostate cancer were investigated and actionable drugs with therapeutic targets were suggested [7].

*SLC45A3* is the second-most common 5′ partner gene in *ERG* rearrangement [8]. *SLC45A3:ERG* (*S:E*) fusion is found in approximately 6% of *ERG* fusion prostate cancer, and concurrent *TMPRSS2* and *SLC45A3* fusions to *ERG* are found in 11% of *ERG* fusion-positive cancer [8]. However, molecular knowledge regarding *S:E* fusion-positive prostate cancers is very limited.

In this study, we aimed to investigate the molecular pathological features of *S:E* fusion-positive prostate cancer and provide potential therapeutic genes and candidate drugs. The study used The Cancer Genome Atlas (TCGA) prostate adenocarcinoma (PRAD) data from the Broad GDAC Firebrowse website. We analyzed cellular pathways through over-representation analysis (ORA), and presented actionable drugs for the patients through the Drug-Target database from Clinical Interpretation of Variants in Cancer (CIViC).

## 2. Materials and Methods

### 2.1. Sample Data Acquisition

TCGA PRAD mRNA expression level data were obtained from the Broad GDAC Firebrowse website (http://gdac.broadinstitute.org/ accessed on 21 August 2021). Additionally, TCGA PRAD patient characteristics including molecular and pathological information such as Age, TNM stage, Gleason Score, PSA, and vital status (Table 1, Tables S1 and S2) was obtained from the Broad GDAC Firebrowse website. 

### 2.2. Case and Control Sample Selection

In this study, *S:E* fusion-positive cancer and *T:E* fusion-positive cancer were used as the case groups, and fusion-negative cancer was used as the control group. The Tumor Fusion Gene Data Portal website (https://tumorfusions.org/ accessed on 21 August 2021) was used to select fusion-positive samples. *S:E* fusion-positive cancers (*n* = 10) were selected after excluding two cases (TCGA-G9-6351-01A, TCGA-YL-A9WX-01A) which have concurrent *T:E* and *S:E* fusions. A fusion of exon 1 of *TMPRSS2* with exon 4/5 of *ERG* were selected as *T:E* fusion-positive cancers (*n* = 86). Fusion-negative cancers (*n* = 50) were selected from a sample of TCGA PRAD data by sorting *ERG* expression level in descending order and selecting the bottom to 50th sample.

### 2.3. Selection of Genes Related to S:E and T:E Fusion

The Pearson correlation test was applied to measure the statistical correlations between gene expression levels of *ERG* and other genes. Correlated genes were obtained by considering genes with an absolute correlation coefficient of over 0.3, based on our previous study [7]. As a result, 1325 genes and 2829 genes were selected from 20,531 gene sets of TCGA PRAD data in *S:E* fusion and *T:E* fusion, respectively. 

### 2.4. Pathway Analysis via ConsensusPathDB (CPDB) and Over-Representation

Each selected gene in *S:E* fusion and *T:E* fusion was used as an input for Over-Representation Analysis (ORA) via ConsensusPathDB (CPDB, http://cpdb.molgen.mpg.de/ accessed on 14 September 2021). Pathways were selected with this criteria: a minimum overlap input list (*n* = 2) and *p*-value cutoff (*p*-value < 0.01), and resulted from databases described by a previous study [9].

### 2.5. Organization of Gene-Drug Network via CIViC 

A clinical drug database was obtained from Clinical Interpretation of Variants in Cancer (CIViC). We performed gene–drug network analysis with each selected gene (|R| > 0.3) in *S:E* fusion, and identified therapeutic target genes and their targeting drugs.

### 2.6. Data Visualization and Analysis via R

R statistical software (version 3.6.3) was used for analysis in this study. Table 1, Tables S1 and S2 were created with the moonBook package in R. Gene selection was performed through a Pearson correlation test in R. Visualization of mRNA expression level was found by the Complexheatmap package in R. Cytoscape 3.8.2 was used to visualize the gene–drug network.

## 3. Results

### 3.1. Clinical Characteristics of Prostate Cancer Patients

**Figure 1:**
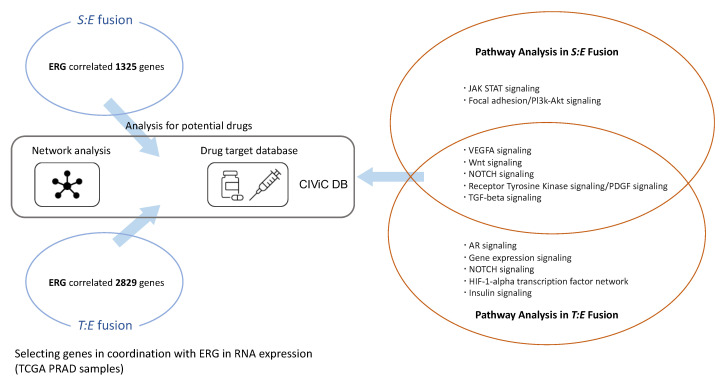
An overview of the analyses in this study. In total, 1325 genes were selected through a Pearson correlation test of *S:E* fusion-positive prostate cancer compared with *ERG* fusion-negative prostate cancer, and 2829 genes were selected through a Pearson correlation test of *T:E* fusion-positive prostate cancer compared with *ERG* fusion-negative prostate cancer. The selected genes were used for pathway analysis using ORA via CPDB, and for gene–drug network analysis via Cytoscape.

First, we obtained RNA-seq data and clinical data of prostate cancer patients from TCGA (Figure 1). The Tumor Fusion Gene Data Portal website was used to identify prostate cancer with structural variation of *ERG*. Ten cases were identified as *S:E* fusion-positive prostate cancer after removing two cases (TCGA-G9-6351-01A, TCGA-YL-A9WX-01A) with concurrent *T:E* and *S:E* fusions. *T:E* fusion-positive prostate cancer was obtained by considering fusions of exon 1 of *TMPRSS2* to exon 4 or exon 5 of *ERG* (*n* = 86) because exon 4 or exon 5 of *ERG* are the most abundant breaking sites, generating *T:E* fusions [10]. *S:E* fusion and *T:E* fusion result in *ERG* overexpression, and thus the *ERG* fusion-negative group was selected by a low expression of *ERG* (*n* = 50). 

Patient characteristics were identified by comparing *S:E* fusion-positive vs. *ERG* fusion-negative patients, *S:E* fusion-positive vs. *T:E* fusion-positive patients, and *T:E* fusion-positive vs. *ERG* fusion-negative patients, and there were no significant differences in PSA, tumor stages, and Gleason score as well as age and race (Table 1, Tables S1 and S2).

### 3.2. Cellular Pathways Associated with S:E Fusion-Positive Prostate Cancer

**Figure 2:**
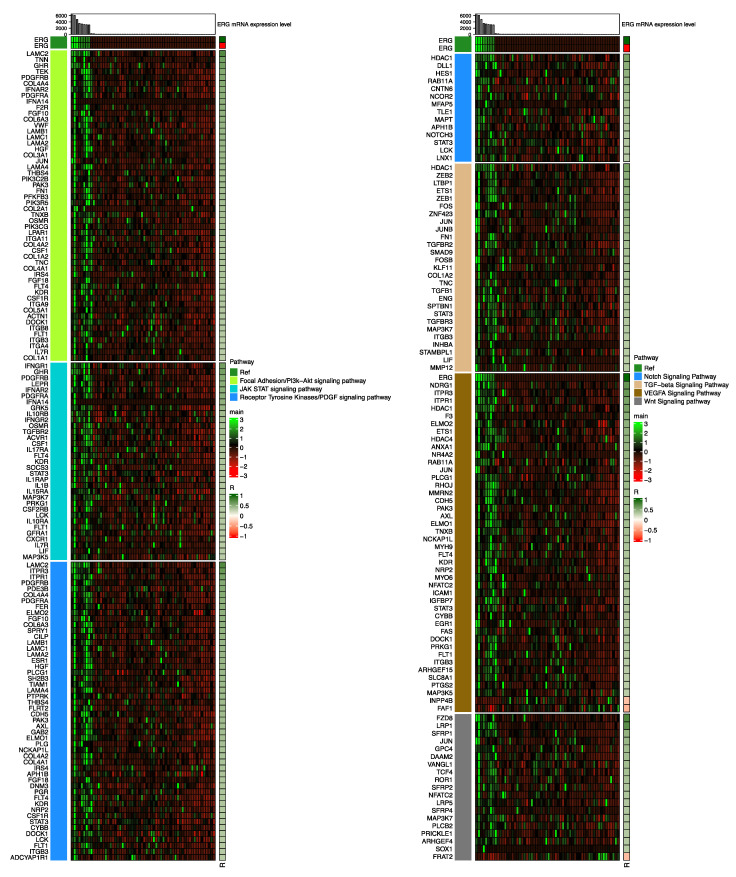
Heatmap for pathways and genes selected by ORA via CPDB in *S:E* fusion. Pathways and genes selected by ORA via CPDB in *S:E* fusion are presented by expression heatmap. The annotation on top represents *ERG* mRNA expression levels of *S:E* fusion-positive patients (*n* = 10) and *ERG* fusion-negative patients (*n* = 50). Rows are gene lists for the selected pathways calculated by a Pearson correlation test. Analysis parameters are described in the Experimental Section.

To investigate cellular signaling pathways related to *S:E* fusion-positive prostate cancer, we identified genes significantly correlated with *ERG* expression in *S:E* fusion-positive prostate cancer via the Pearson correlation test. To minimize false positive results, we used an absolute value of correlation coefficient 0.3 as the minimal level, as proved previously [7], and 1325 genes were identified (Figure 1). Among the 1325 genes, 1244 genes were positively correlated with *ERG* expression while 81 genes were negatively correlated. *FZD8* showed the highest correlation with *ERG* expression (Table S3), and it is consistent with previous studies [11,12]. *FZD8* is directly targeted and activated by *ERG*, which is involved in bone metastasis of prostate cancer by regulating Wnt-11 and TGF-β signaling to stimulate epithelial–mesenchymal transition in prostate cancer [11,12]. The selected 1325 genes were further applied to ConsensusPathDB (CPDB) to analyze cellular signaling pathways, and seven cancer-related pathways, the focal adhesion kinase (FAK)/PI3K-Akt signaling pathway, JAK-STAT pathway, Notch signaling pathway, receptor tyrosine kinase/PDGF signaling pathway, TGF-β signaling pathway, VEGFA signaling pathway, and Wnt signaling pathway were identified with 163 genes (Table S3). Interestingly, genes in these cancer-related pathways showed an overall positive correlation with *ERG* expression (Figure 2). Only three genes, *FRAT2* in the Wnt/β-catenin signaling pathway (*p*-value = 0.007) and *FAF1* and *INPP4B* in the VEGFA signaling pathway (*p*-value = 0.00001), showed a negative correlation to *ERG* (Figure 2). *STAT3* was most frequently found in *S:E*-specific cancer pathways, such as the JAK-STAT pathway, Notch signaling pathway, receptor tyrosine kinase/PDGF signaling pathway, TGF-β signaling pathway, and VEGFA signaling pathway (Table S3). *HDAC1*, *JUN*, *PDGFRA*, and *PDGFRB* were found in multiple signaling pathways including the VEGFA signaling pathway, TGF-β signaling pathway, focal adhesion kinase (FAK)/Pl3k-Akt signaling pathway, JAK-STAT pathway, receptor tyrosine kinase/PDGF signaling pathway, and Wnt signaling pathway (Table S3).

### 3.3. Cellular Pathways Associated with T:E Fusion-Positive Prostate Cancer

We previously studied the signaling pathways involved in *T:E* fusion-positive cancer using *T:E* fusion-positive cases selected based on *ERG* expression level [7]. However, this time, we focused more on the fusion of exon 1 of *TMPRSS2* to exon 4/5 of *ERG*, which is the most abundantly found rearrangement in *T:E* fusion-positive cancer, as a way to solidly establish *T:E* fusion-positive cancer. Then, genes associated in *T:E* fusion-positive cancers were examined by the same criteria used for analysis of *S:E* fusion-positive cancers, and 2829 genes were obtained. The selected 2829 genes were used for ORA via CPDB, and nine pathways, the androgen receptor signaling pathway, gene expression signaling pathway, HIF-1-alpha transcription factor pathway, insulin signaling pathway, Notch signaling pathway, receptor tyrosine kinase signaling pathway, TGF-β signaling pathway, VEGFA signaling pathway, and Wnt signaling pathway, were identified with 451 genes (Table S4, Figure S1). These signaling pathways were found to be very similar to those in our previous study, even though different *T:E* fusion-positive cancer samples were used (Figure S2A) [7].

### 3.4. Comparison of Cellular Pathways Involved in S:E Fusion-Positive Prostate Cancer vs. T:E Fusion-Positive Prostate Cancer

To identify unique cellular pathways in *S:E* fusion-positive cancer, we compared signaling pathways between *S:E* fusion-positive cancer and *T:E* fusion-positive cancer. Interestingly, five pathways, the Notch signaling pathway, receptor tyrosine kinase/PDGF signaling pathway, TGF-β signaling pathway, VEGFA signaling pathway, and Wnt signaling pathway, were in common in both *S:E* fusion-positive and *T:E* fusion-positive prostate cancers (Figure S2B). Two pathways, the focal adhesion kinase (FAK)/Pl3K-Akt signaling pathway and JAK-STAT signaling pathway, were uniquely identified in *S:E* fusion-positive prostate cancer. We applied different cut-off values of |R| from the Pearson correlation test to exclude the possibility of false-positive results caused by the different number of samples in *S:E* fusion-positive and *T:E* fusion-positive prostate cancer. Surprisingly, however, the focal adhesion kinase (FAK)/Pl3K-Akt signaling pathway and JAK-STAT signaling pathway were significantly enriched in *S:E* fusion-positive prostate cancer.

### 3.5. Candidate Target Genes and Drugs

**Figure 3:**
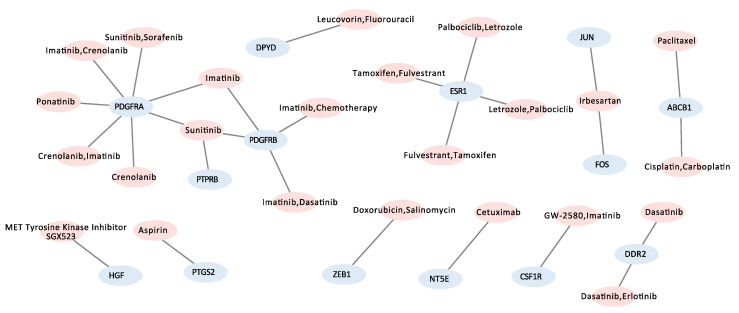
Visualization for gene–drug network analysis. Gene–drug network analysis was performed using the selected 1325 genes and the CIViC drug database via Cytoscape. Genes are represented in blue and drugs are represented in pink.

Next, we investigated specific therapeutic targets and candidate actionable drugs for *S:E* fusion-positive prostate cancer by performing gene–drug network analysis. In total, there were 1325 genes serving as the input, and the CIViC database and Cytoscape software program were used; 24 drugs targeting 14 genes were found in the *S:E* fusion-positive group (Figure 3, Table 2). *PDGFRA* and *PDGFRB* were targeted by multiple drugs, and imatinib, a BCR-ABL inhibitor [13], was identified to target both *PDGFRA* and *PDGFRB* in *S:E* fusion-positive prostate cancer. *PDGFRA* and *PDGFRB* have 0.49 and 0.53 of the correlation coefficiency for *ERG* expression, respectively, in *S:E* fusion-positive cancer. However, these genes were not found in *T:E* fusion used in the current study (Tables S3 and S4). This suggests a significant function of *PDGFRA* and *PDGFRB* in *S:E* fusion-positive cancer.

## 4. Discussion

As a well-known oncogene, the rearrangement of *ERG* is often caused by fusion with other androgen-related 5′ partner genes such as *TMPRSS2*, *SLC45A3*, and *NDRG1*. These rearrangements have a significant influence on *ERG* expression level, which could provide clues for targeted therapy of prostate cancer patients [14]. However, targeted drugs for *ERG* are not available to date, and detailed molecular features of these fusion types based on fusion partners have not been extensively studied yet. With publicly accessible TCGA mRNA expression data, we first examined molecular signaling pathways associated with *S:E* fusion-positive or *T:E* fusion-positive patients. The Pearson correlation test was applied and |R| > 0.3 was used to obtain significantly associated genes in each group of patients. The cutoff of |R| > 0.3 was previously tested to examine the false-positive ratio [7], and more than 98.8475% of genes showed true positive results, suggesting that the cutoff of |R| > 0.3 is reasonable to apply in the current study. By analyzing molecular signaling pathways, we identified seven cancer-specific pathways; the focal adhesion kinase (FAK)/PI3K-Akt signaling pathway, JAK-STAT pathway, Notch signaling pathway, receptor tyrosine kinase/PDGF signaling pathway, TGF-β signaling pathway, VEGFA signaling pathway, and Wnt signaling pathway are significantly altered in the *S:E* fusion-positive group. Moreover, the autophagy signaling pathway, which plays an important role in prostate cancer [15], was altered in the *S:E* fusion-positive group (Figure S3). Most genes with autophagy-associated pro-survival roles in cancer under this signal are highly expressed in *S:E* fusion-positive prostate cancer compared with *S:E* fusion-negative cancer. In relation to this, the protein encoded from the *FAF1* gene induces apoptosis by binding to the *FAS* antigen and acts as a tumor suppressor involved in the regulation of NF-kB signaling [16], and the *INPP4B* gene acts as a suppressor for invasion of prostate carcinoma PC-3 cells and oncogenic PKC signaling [17]. These two genes were negatively correlated with the *S:E* fusion-positive group (Figure 2). *FRAT2,* a regulator of the Wnt signaling pathway [18], was negatively correlated with the *S:E* fusion-positive group, but it was significantly highly expressed compared with normal prostate tissue (Figure S4), suggesting that it might be a good therapeutic target for prostate cancer but not for the *S:E* fusion-positive group specifically.

Furthermore, we investigated cellular signaling pathways unique to *S:E* fusion-positive prostate cancer by comparing cellular signaling pathways to those altered in *T:E* fusion-positive prostate cancers. Remarkably, the focal adhesion kinase (FAK)/Pl3K-Akt signaling pathway and JAK-STAT signaling pathway were uniquely identified in *S:E* fusion-positive prostate cancer (Figure 1). *ERG* rearrangements result in *ERG* overexpression; thus, we previously thought that cellular signaling pathways both in *S:E* fusion-positive prostate cancer and *T:E* fusion-positive prostate cancer were alike. However, as shown in the current study, there are unique pathways for *S:E* fusion-positive prostate cancer and *T:E* fusion-positive cancer. Genetic or epigenetic defects destabilize genomes and, in turn, induce genomic rearrangement. Thus, there are high chances of having different molecular and cellular features between *S:E* fusion-positive cancer cells and *T:E* fusion-positive cancer cells, which was shown in our current study. We further searched whether specific DNA cleavage mechanisms exist to induce *S:E* fusion or *T:E* fusion. Previously, there were three concepts that describe DNA break in prostate cancer. First, androgen promotes DNA cleavage in both regions of *TMPRSS2* and *ERG*, and mediates *T:E* translocation by recruiting topoisomerase 2 beta (TOP2B) and AR to the specific sites [19]. In addition, sh*TOP2B*-expressing cells inhibit *TMPRSS2* gene expression. Considering that *SLC45A3* is transcribed in an AR-dependent manner and sh*TOP2B*-expressing cells inhibit *SLC45A3* gene expression, TOP2B may be one of mechanisms to cause a break in *SLC45A3* and *ERG* and induce *S:E* fusion. In addition, androgen and genotoxic stress recruit cytidine deaminase (AID) and LINE1-encoded ORF2 endonuclease to the specific sites and induce DNA cleavage of *TMPRSS2*, *SLC45A3*, *ERG*, and *ETV1* in prostate cancer [20]. Another study identified that the depletion of *SPOP* increases topoisomerase 2 alpha (TOP2A) in DNA, and in turn removes tyrosyl-DNA phosphodiesterase 1(TDP1), tyrosyl-DNA phosphodiesterase 2(TDP2), and MRE11 from DNA, which triggers DNA damage and break [21]. To explore *S:E* fusion mechanism based on this concept, gene expression of *SPOP, TOP2A, TDP1, TDP2*, and *MRE11* was examined, but no significant differences were found between the *S:E* fusion positive- and negative- groups (Figure S5). Further biochemical experiments are certainly needed to investigate DNA cleavage mechanisms to promote *S:E* fusion. 

Prostate cancer is the second most common cancer causing mortality in males. Fortunately, cancer driver genes in prostate cancer, *ERG*, *ETV1*, *ETV4*, *SPOP*, and *FOXA1*, have been identified and their roles have been proved through basic and clinical research [2,14,22,23]. However, treatment methods for each type of prostate cancer have not been established. Thus, in this study, we applied a drug repurposing strategy to identify and suggest therapeutic targets and candidate drugs in *S:E* fusion-positive prostate cancer. In total, 1325 genes were utilized to perform gene–drug network analysis, and interestingly, imatinib targeted *PDGFRA* and *PDGFRB. PDGFRA* and *PDGFRB* were significantly enriched and correlated with *ERG* expression in the *S:E* fusion-positive group (Figure S6) but not in the *T:E* fusion-positive group used in the current study (Tables S3 and S4). In addition, previous studies show that *PDGFR*s can play a role in activation of JAK-STAT and focal adhesion pathways [24,25], which are pathways unique to *S:E* fusion-positive prostate cancer. *PDGFRA* and *PDGFRB* are genes involved in the focal adhesion kinase (FAK)/PI3K-Akt signaling pathway, JAK-STAT pathway, and receptor tyrosine kinase/PDGF signaling pathway in *S:E* fusion-positive prostate cancer. This suggests that *PDGFRA* and *PDGFRB* might be effective therapeutic targets, and imatinib could be a potential candidate drug for *S:E* fusion-positive prostate cancer. In a previous study, imatinib did not have beneficial effects on prostate cancer [26], and this may have been due to a failure in patient selection. Our study suggests that imatinib may only be effective for *S:E* fusion-positive prostate cancer. In addition, imatinib was proposed as an anti-cancer targeted drug by targeting and suppressing *PDGFR* in breast cancer [27]. *PDGFR*s are expressed for the bone metastasis of prostate cancer cells [28]. *FZD8,* the highest *ERG*-correlated gene (R = 0.761) in *S:E* fusion-positive prostate cancer (Table S3, Figure S6), is involved in bone metastasis in prostate cancer [12]. *FZD8* was significantly regulated by imatinib treatment in gastrointestinal stromal tumors [29]. These findings suggest that *S:E* fusion-positive prostate cancer might be more favorable for bone metastasis, and downregulation of *PDGFR* and *FZD8* by imatinib may protect from bone metastasis, which is a topic for future study.

## 5. Conclusions

To conclude, we investigated the molecular features defined by different fusion partners of *ERG* in prostate cancer. The focal adhesion kinase (FAK)/PI3K-Akt signaling pathway and JAK-STAT pathway were characterized as cellular signaling pathways unique to *S:E* fusion-positive prostate cancer. In addition, imatinib, targeting *PDGFRA* and *PDGFRB*, was suggested as a potent actionable drug specific for *S:E* fusion-positive prostate cancer. Despite the obvious limitations of in silico analysis, as a first step, our study provides a rigorous method to understand the molecular characteristics of a certain type of cancer, and suggests possible therapeutic targets and candidate drugs. Further biochemical experiments will certainly necessary to validate our in silico analysis, and moreover investigate the underlying molecular mechanism and function of *S:E* fusion in prostate cancer. 

## Figures and Tables

**Table 1:** Patient characteristics of *S:E* fusion-positive and fusion-negative patients.

	*SLC45A3—ERG*-Negative	*SLC45A3—ERG*-Positive	*p*-Value
	(*N* = 50)	(*N* = 10)	
Age	61.6 ± 7.4	58.7 ± 7.7	0.269
M stage			1
- M0	45 (97.8%)	8 (100.0%)	
- M1b	1 (2.2%)	0 (0.0%)	
T stage			0.884
- T1c	19 (46.3%)	4 (66.7%)	
- T2a	5 (12.2%)	1 (16.7%)	
- T2b	4 (9.8%)	0 (0.0%)	
- T2c	4 (9.8%)	0 (0.0%)	
- T3a	5 (12.2%)	1 (16.7%)	
- T3b	3 (7.3%)	0 (0.0%)	
- T4	1 (2.4%)	0 (0.0%)	
Gleason Score			0.474
- 6	6 (12.0%)	2 (20.0%)	
- 7	25 (50.0%)	7 (70.0%)	
- 8	7 (14.0%)	1 (10.0%)	
- 9	11 (22.0%)	0 (0.0%)	
- 10	1 (2.0%)	0 (0.0%)	
Laterality			0.324
- Bilateral	43 (86.0%)	7 (70.0%)	
- Left	1 (2.0%)	0 (0.0%)	
- Right	6 (12.0%)	3 (30.0%)	
Vital Status			1
- LIVING	48 (96.0%)	10 (100.0%)	
- DECEASED	2 (4.0%)	0 (0.0%)	
PSA level	9.7 ± 52.3	0.1 ± 0.0	0.262
Race			
- WHITE	9 (100.0%)	4 (100.0%)	

**Table 2:** List of 14 target candidate genes via gene-drugs network based on the CIViC database.

Target Gene List	Actionable Drugs	Related Pathway
**CSF1R**	GW-2580, Imatinib	Focal adhesion kinase (FAK)/Pl3k-Akt signaling pathway,
		Receptor tyrosine kinase/PDGF signaling pathway
**ESR1**	Fulvestrant, Tamoxifen, Letrozole, Palbociclib	Receptor tyrosine kinase/PDGF signaling pathway
**FOS**	Irbesartan	TGF-beta signaling pathway
**HGF**	MET tyrosine kinase inhibitor SGX523	Focal adhesion kinase (FAK)/Pl3k-Akt signaling pathway,
		Receptor tyrosine kinase/PDGF signaling pathway
**JUN**	Irbesartan	Focal adhesion kinase (FAK)/Pl3k-Akt signaling pathway,
		VEGFA signaling pathway, Wnt signaling pathway, TGF-beta signaling pathway
**PDGFRA**	Crenolanib, Imatinib, Sunitinib, Sorafenib, Ponatinib	Focal adhesion kinase (FAK)/Pl3k-Akt signaling pathway,
		Receptor tyrosine kinase/PDGF signaling pathway, JAK STAT signaling pathway
**PDGFRB**	Sunitinib, Dasatinib, Imatinib, Chemotherapy	Focal adhesion kinase (FAK)/Pl3k-Akt signaling pathway,
		JAK STAT signaling pathway, Receptor tyrosine kinase/PDGF signaling pathway
**PTGS2**	Aspirin	VEGFA signaling pathway
**ZEB1**	Doxorubicin, Salinomycin	TGF-beta signaling pathway
**ABCB1**	Paclitaxel, Cisplatin, Carboplatin	NA
**DDR2**	Dasatinib, Erlotinib	NA
**DPYD**	Leucovorin, Fluorouracil	NA
**NT5E**	Cetuximab	NA
**PTPRB**	Sunitinib	NA

## Data Availability

TCGA PRAD mRNA expression level data is available in the Broad GDAC Firebrowse website (http://gdac.broadinstitute.org/ (accessed on 8 August 2022)).

## Supplementary Materials

The following supporting information can be downloaded at: https://www.mdpi.com/article/10.3390/jpm12111818/s1, Figure S1: Gene expression heatmap for selected pathways in *T:E* fusion-positive prostate cancer and *T:E*-negative prostate cancer. The annotation on top represents *ERG* mRNA expression levels of *T:E* fusion-positive patients (*n* = 86) and *T:E* fusion-negative patients (*n* = 50). Rows represent genes for nine selected pathways. Analysis parameters are described in the Experimental Section. Figure S2: (A) Venn diagram for pathway lists to show overlap between a previous *T:E* fusion study and our *T:E* fusion study. (B) Venn diagram for pathway lists to show overlap between *S:E* fusion and *T:E* fusion. Figure S3: Gene expression heatmap for autophagy-related genes in TCGA PRAD data. The annotation on top represents *ERG* mRNA expression levels in TCGA PRAD data. The R-value calculated by the Pearson correlation test is shown on the right. Figure S4: Box plot for two genes (*ERG*, *FRAT2*) in *S:E* fusion-positive prostate cancer tissue (*n* = 10) and normal prostate tissue (*n* = 52). Figure S5: Box plot for three genes (*ERG, SPOP, TOP2A, TDP1, TDP2* and *MRE11*) in *S:E* fusion-positive prostate cancer (*n* = 10) and *ETS(ERG, ETV1, ETV4)* fusion-negative prostate cancer (*n* = 12). Figure S6: Gene expression heatmap for four genes (*ERG*, *PDGFRA, PDGFRB, FZD8*) in TCGA PRAD data. The annotation on top represents *ERG* mRNA expression levels in TCGA PRAD data. The R-value calculated by the Pearson correlation test is shown on the right. Table S1: Patient characteristics between *S:E* fusion positive and *T:E* fusion positive patient. Table S2: Patient characteristics between *T:E* fusion positive and *T:E* fusion negative patient. Table S3: Gene list with correlation efficiency for selected pathways though *S:E* fusion ORA. Table S4: Gene list with correlation efficiency for selected pathways though *T:E* fusion ORA.
