# Supplementary figures and images for: Systematic Analysis of Cellular Signaling Pathways and Therapeutic Targets for SLC45A3:ERG Fusion-Positive Prostate Cancer
Source: J Pers Med. 2022 Nov 2;12(11):1818. doi: 10.3390/jpm12111818 (PMC9693845; doi:10.3390/jpm12111818)

Supplementary Figure S1.

(A)

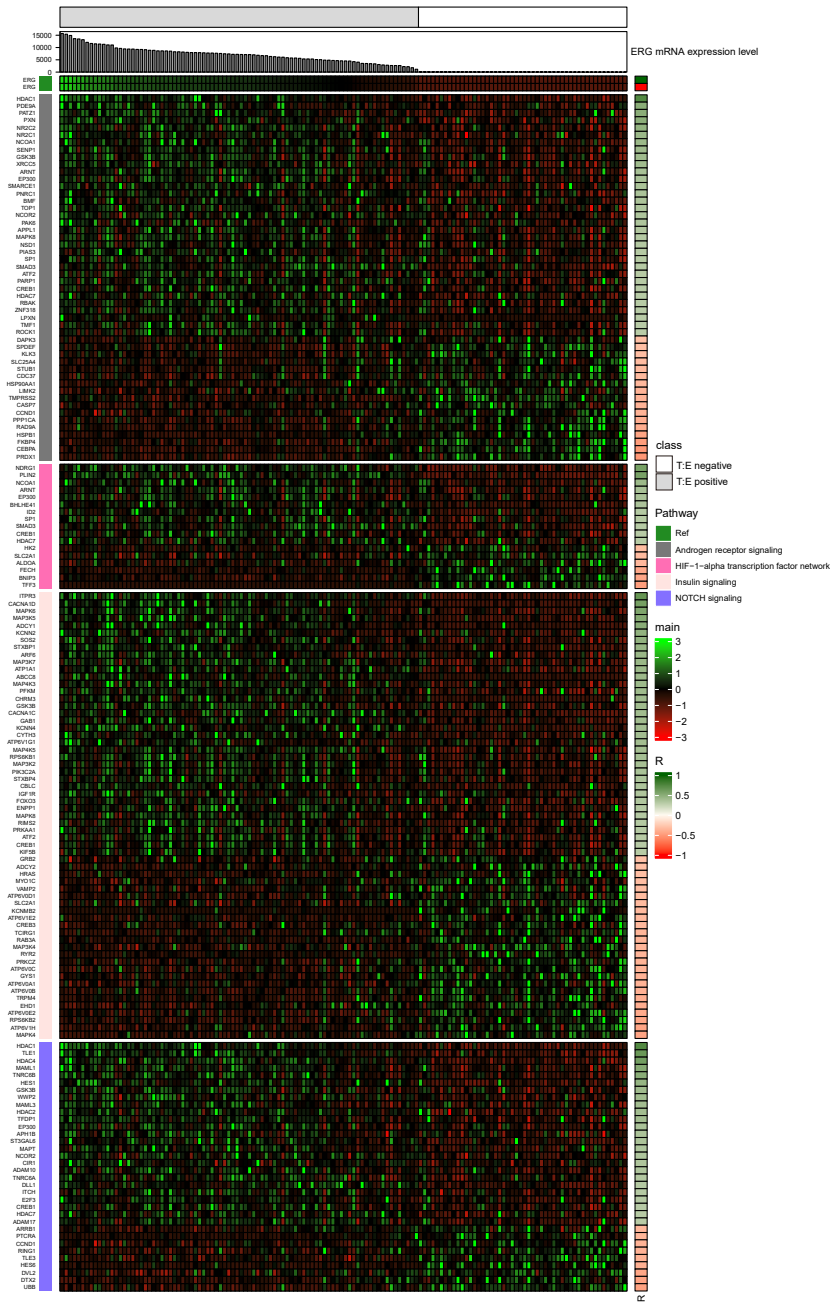

(B)

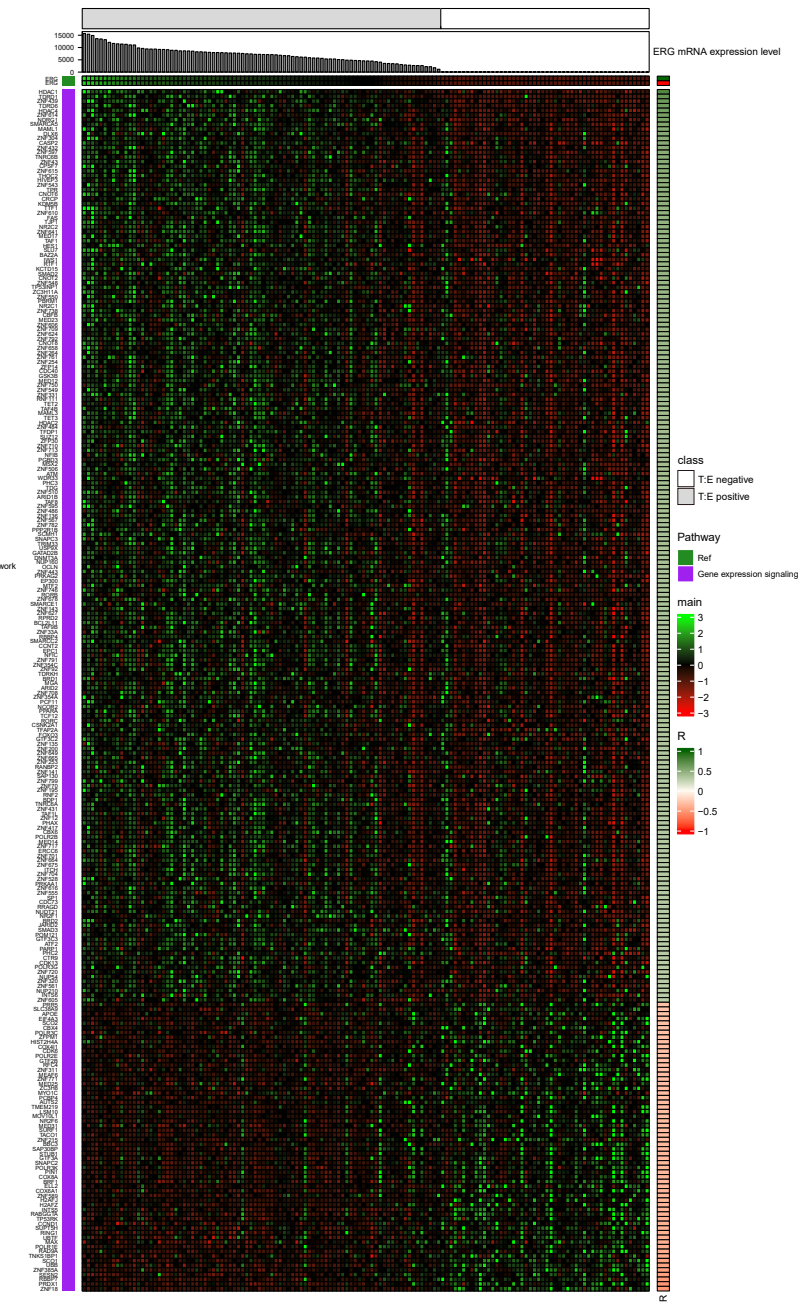

(C)

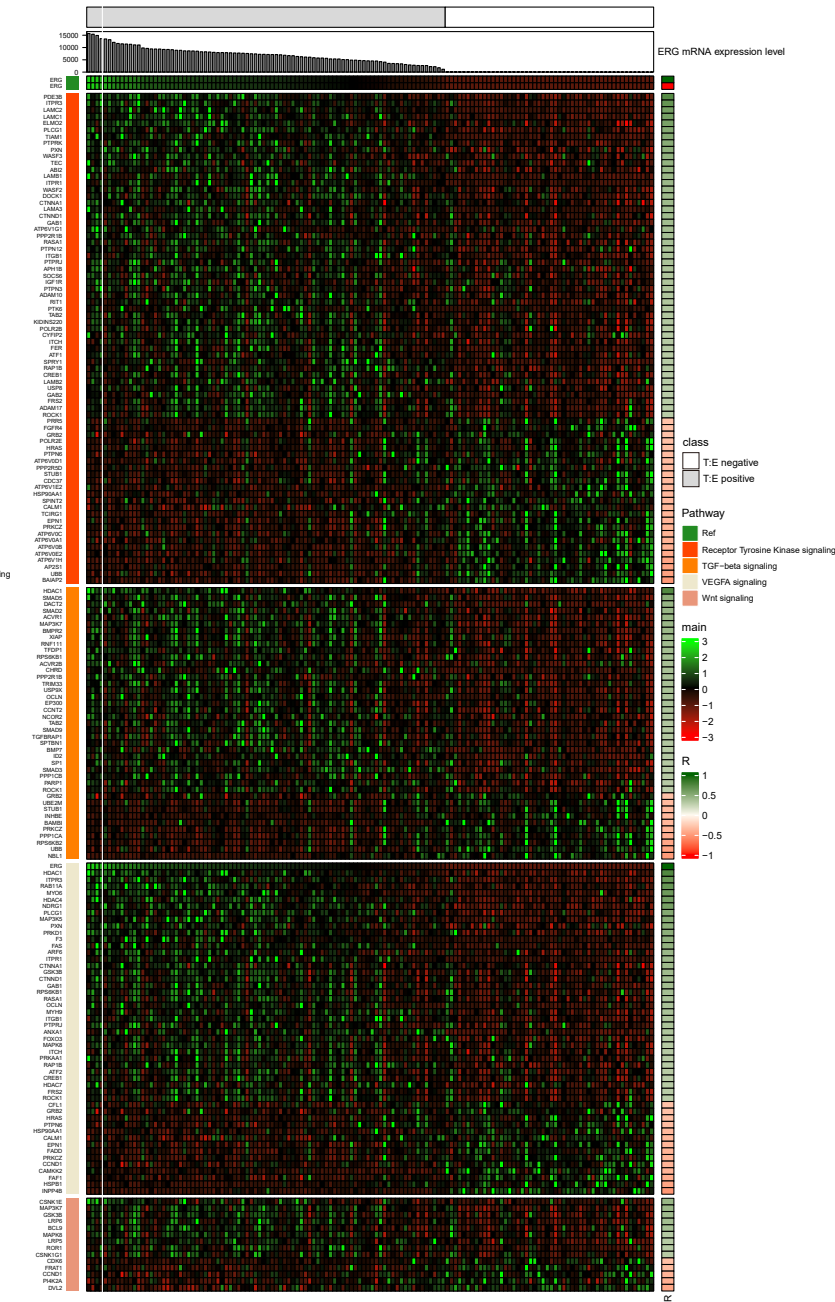

Supplement: Supplementary file 1 [file jpm-12-01818-s001.zip › Figure S1.pdf]

Supplementary Figure S2.

(A)

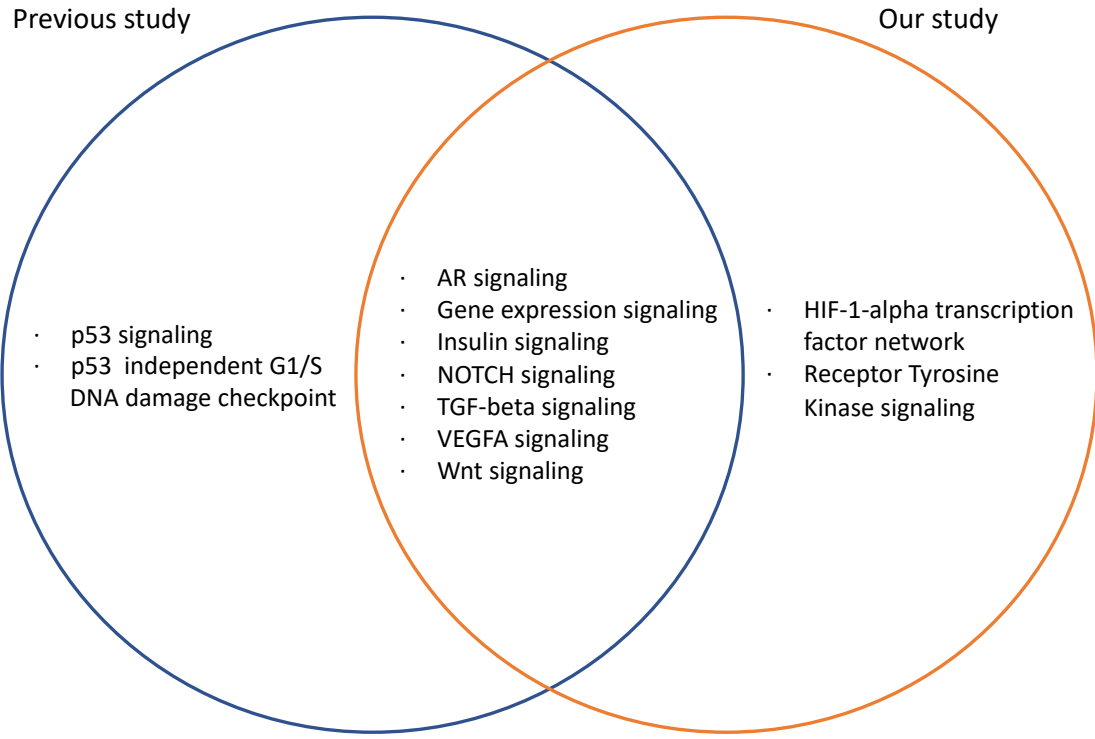

(B)

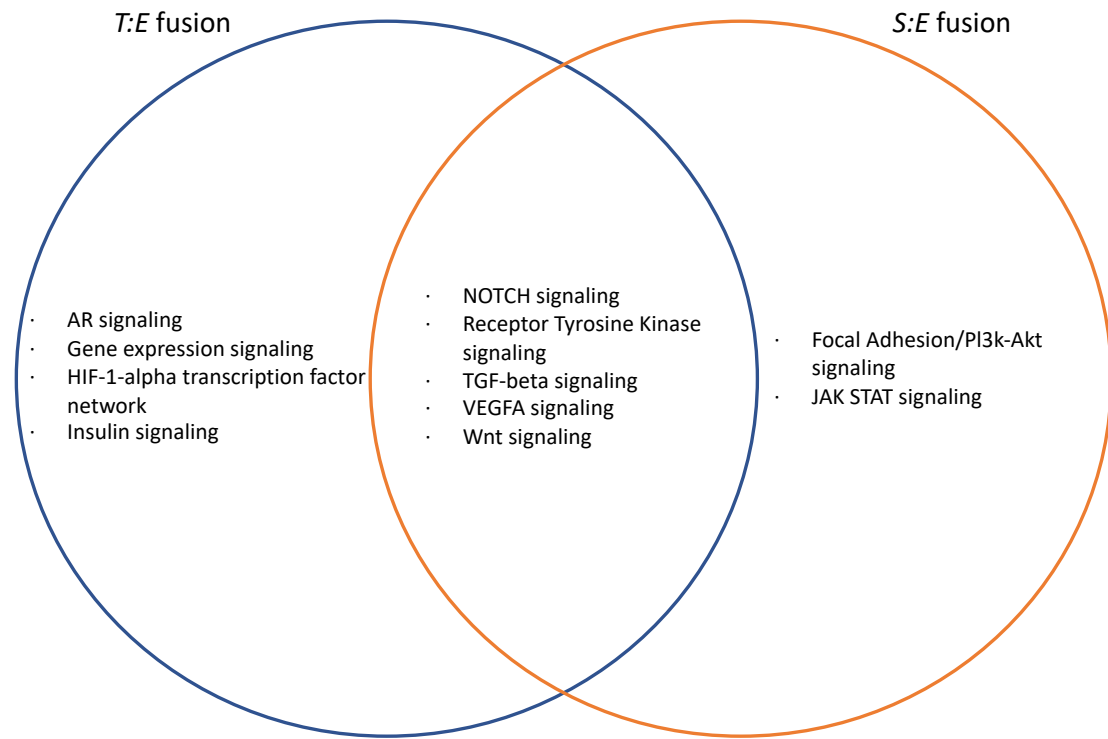

Supplement: Supplementary file 1 [file jpm-12-01818-s001.zip › Figure S2.pdf]

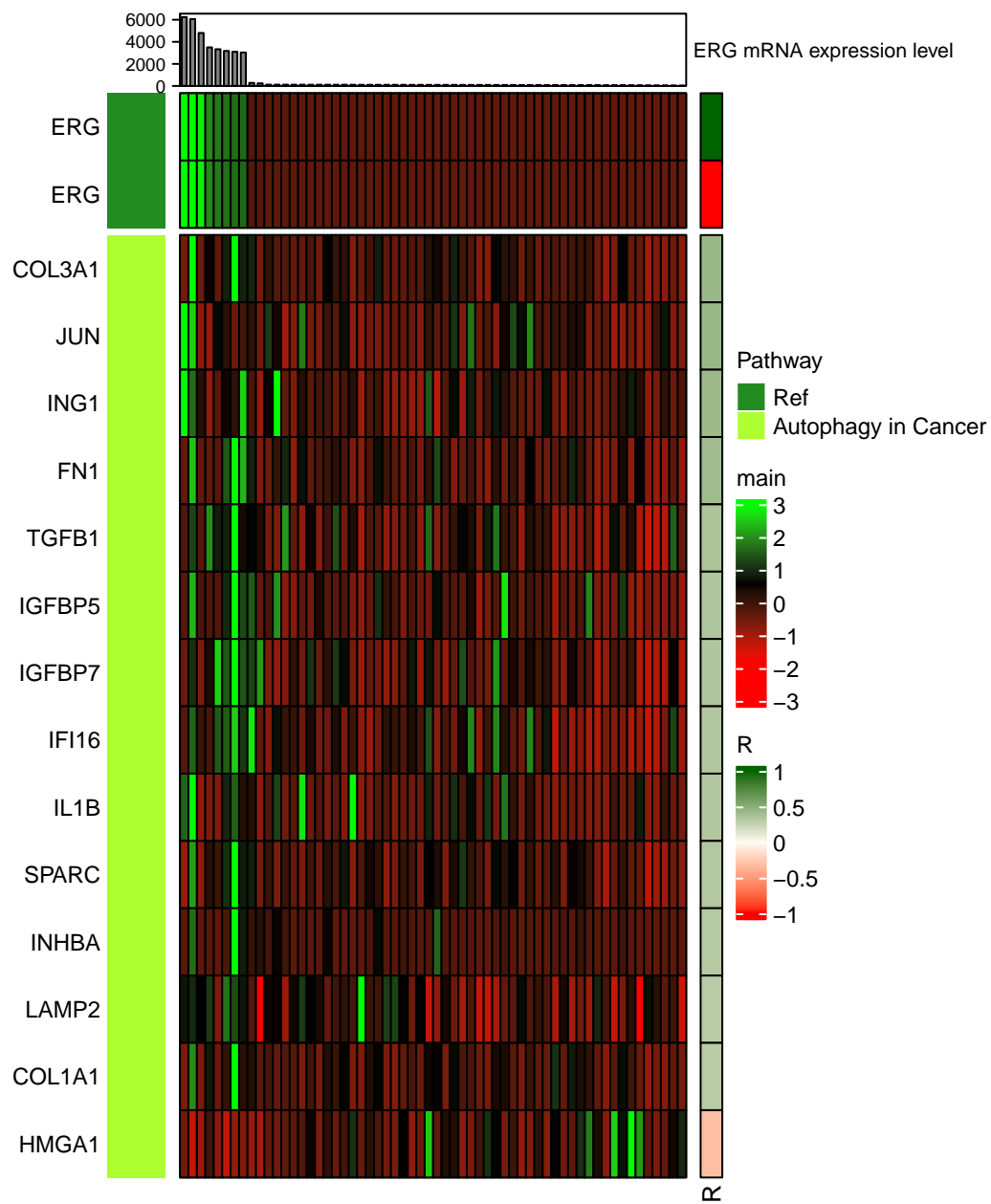

Supplement: Supplementary file 1 [file jpm-12-01818-s001.zip › Figure S3.pdf]

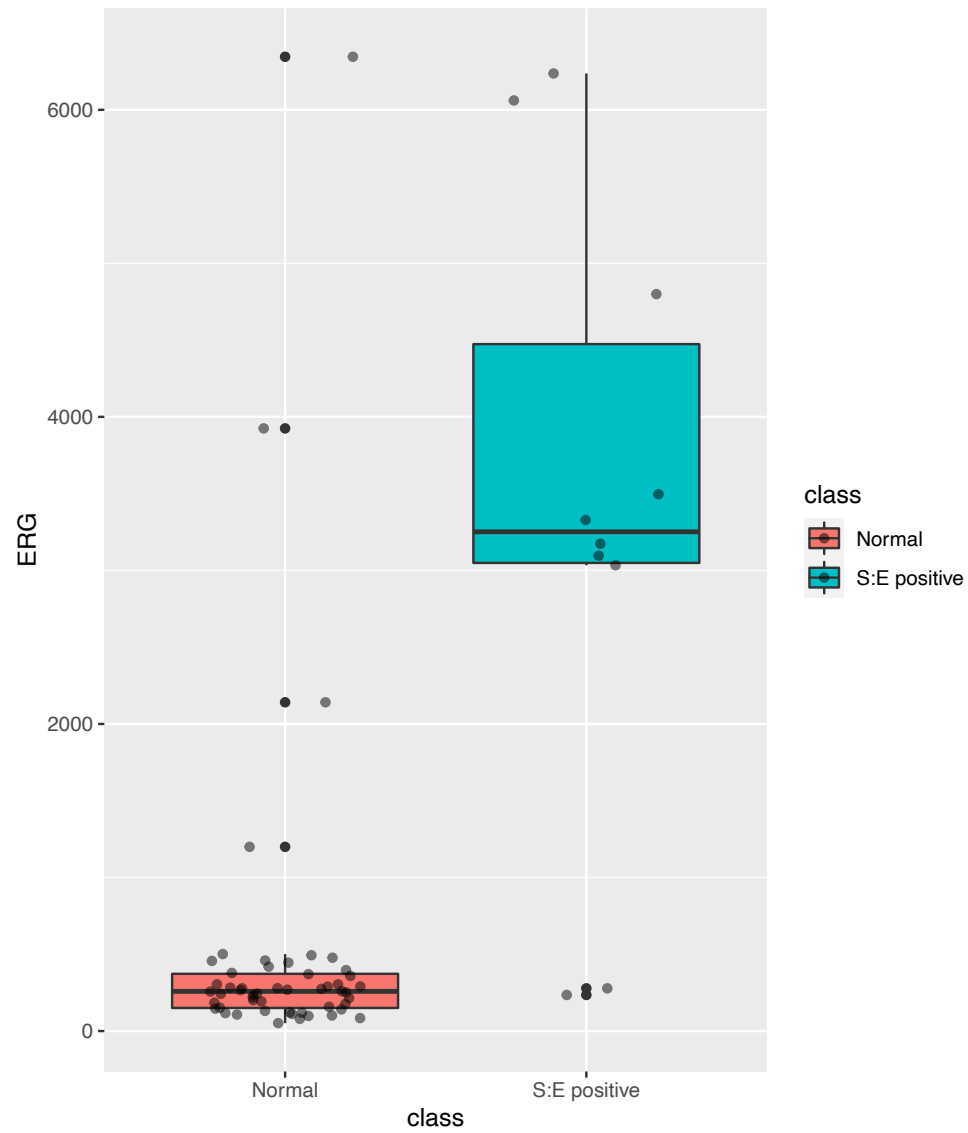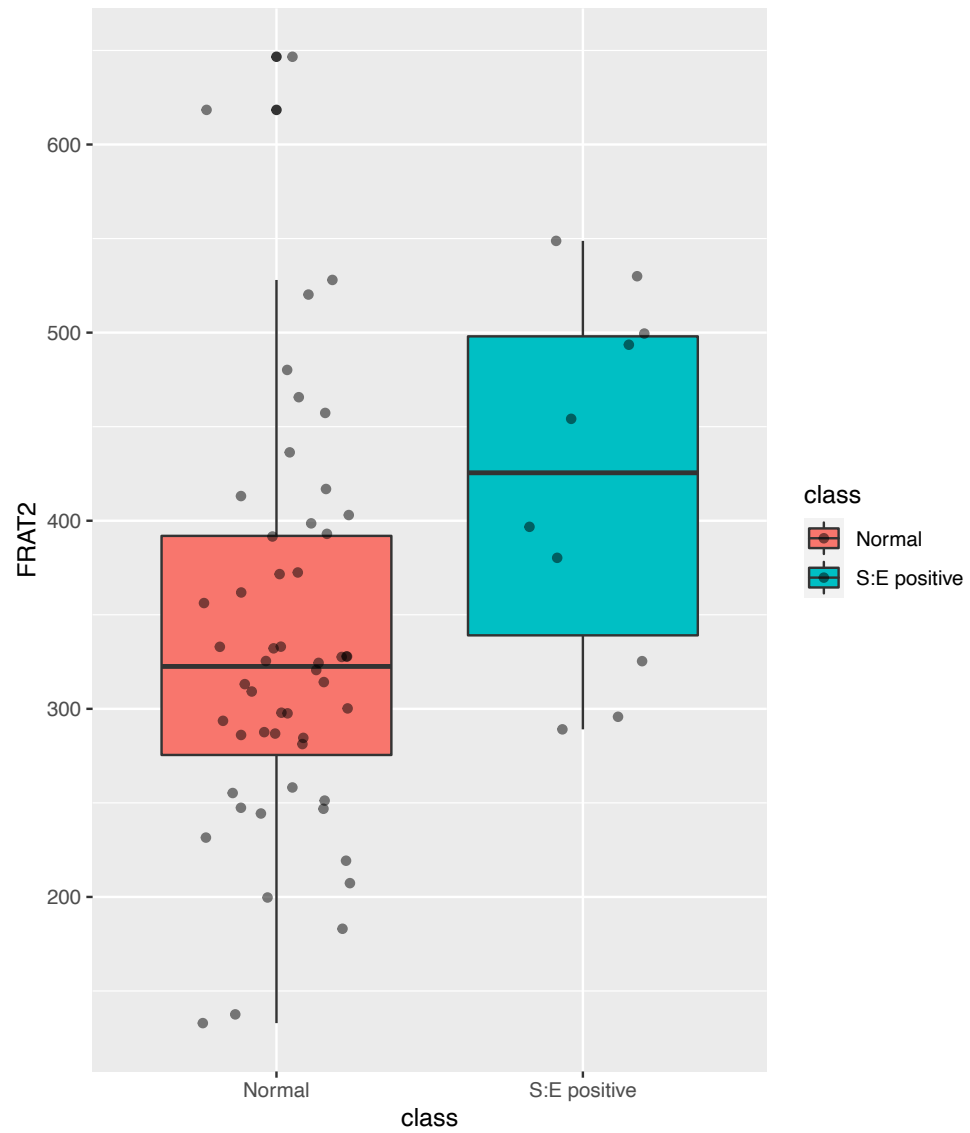

Supplement: Supplementary file 1 [file jpm-12-01818-s001.zip › Figure S4.pdf]

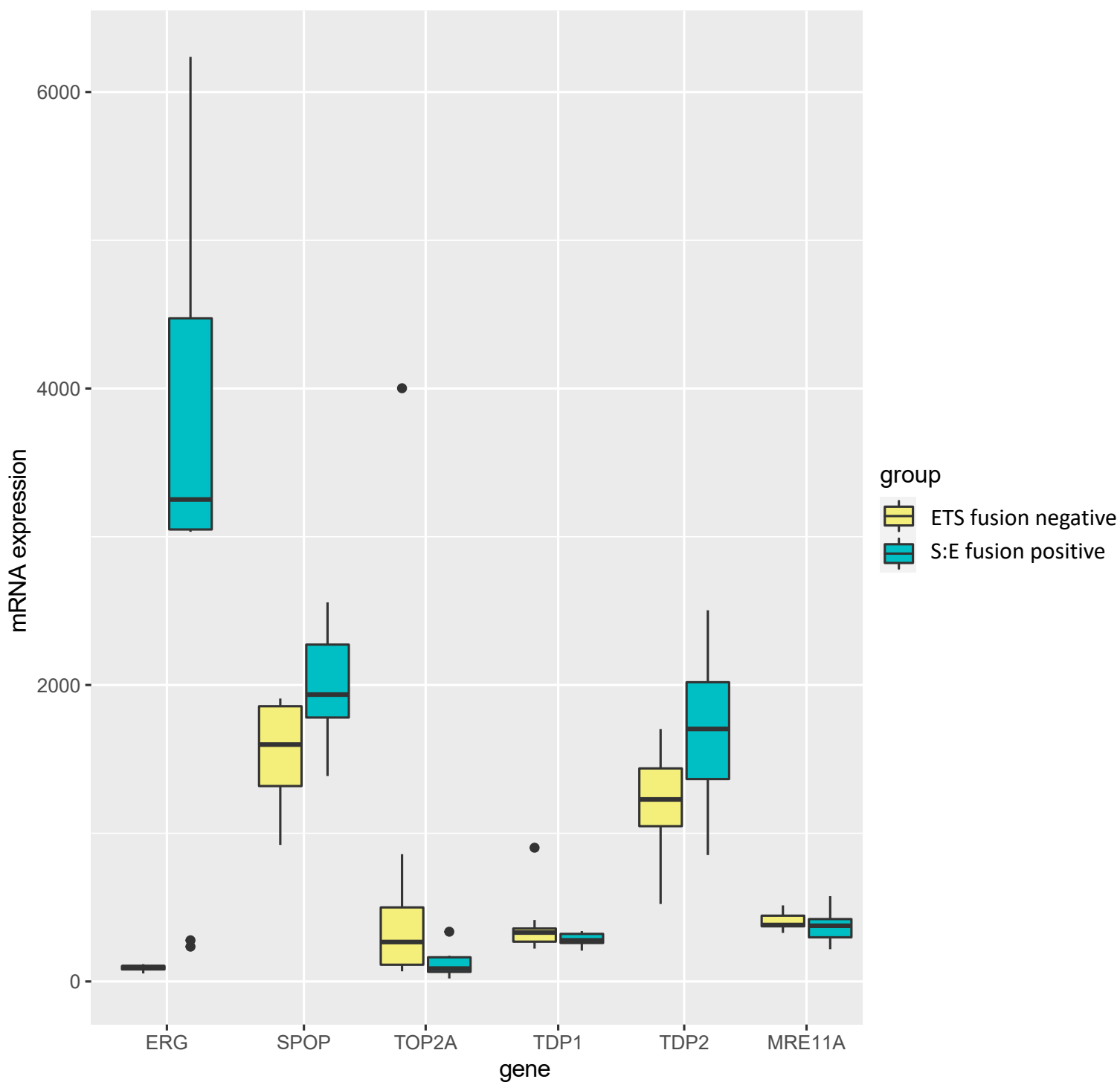

Supplement: Supplementary file 1 [file jpm-12-01818-s001.zip › Figure S5.pdf]

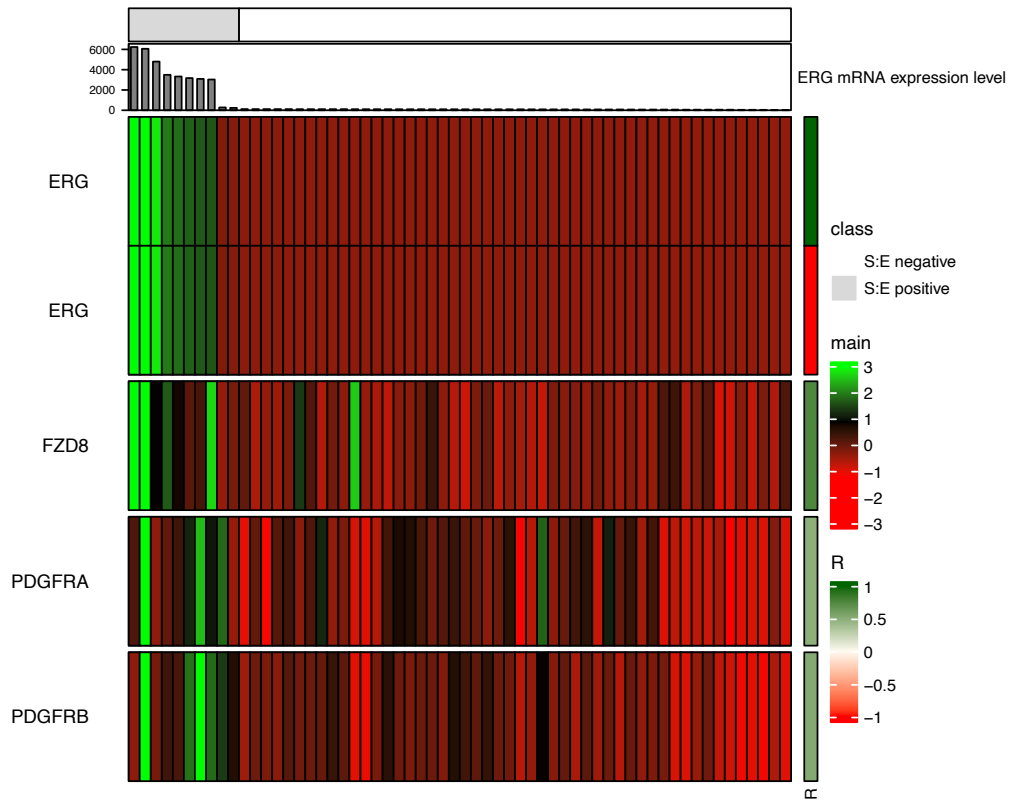

Supplement: Supplementary file 1 [file jpm-12-01818-s001.zip › Figure S6.pdf]
